# A Case of Polyarteritis Nodosa Associated with Cytomegalovirus Infection

**DOI:** 10.1155/2014/604874

**Published:** 2014-11-12

**Authors:** Maiko Kouchi, Shinji Sato, Masahiro Kamono, Akiko Taoda, Kazuyuki Iijima, Atsushi Mizuma, Ruriko Kitao, Masatoshi Mihara, Hideki Ozawa, Tadayuki Ishihara, Atsushi Takagi, Yasuo Suzuki

**Affiliations:** ^1^Division of General Internal Medicine, Department of Internal Medicine, Tokai University School of Medicine, Isehara 259-1193, Japan; ^2^Division of Neurology, Department of Internal Medicine, Tokai University School of Medicine, Isehara 259-1193, Japan; ^3^Division of Rheumatology, Department of Internal Medicine, Tokai University School of Medicine, Isehara 259-1193, Japan; ^4^Department of Neurology, National Hakone Hospital, Odawara 250-0032, Japan

## Abstract

A 77-year-old man suffering from prolonged fever of unknown origin and bilateral leg edema was referred to our hospital. On physical examination, he had fever, general fatigue, bilateral lower leg edema, and muscle weakness of the right upper extremity and left lower extremity. Neurological examination indicated motor and sensory disturbance. Electromyography revealed mononeuritis multiplex and myopathy. A biopsy of the left biceps muscle indicated necrotizing vasculitis with fibrinoid necrosis. Considering all the data together, he was diagnosed as having polyarteritis nodosa (PAN) and concurrent active cytomegalovirus (CMV) infection. His symptoms improved promptly on treatment with 50 mg of prednisolone. This case emphasizes the importance of CMV infection as one of possible etiologies of PAN and reports a therapeutic strategy for this syndrome.

## 1. Introduction 

Polyarteritis nodosa (PAN) is a form of systemic vasculitis that mainly affects medium-sized arteries [1]. It is well recognized that viral infection is one of the pathogenetictriggers of PAN. Hepatitis B infection is well known as a significant etiologic agent in this respect [2]. Cytomegalovirus (CMV) infection is also assumed to play an important role in thepathogenesis of PAN, although the frequency of thisseems to be quite low [3]. Here, we report a case of PAN with active CMV infection. The patient's chief symptoms were prolonged fever and gait disturbance due to peripheral neuropathy and myopathy. It was notable that treatment with prednisolone alone resulted in rapid improvement of theclinical features.

## 2. Case Report 

**Figure 1:**
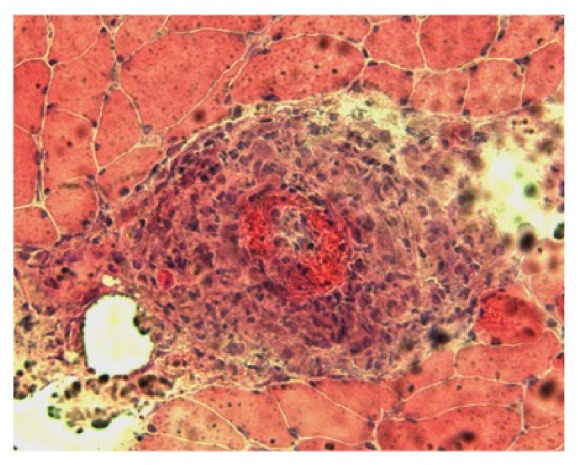
Biopsy findings of left biceps muscle. Inflammatory cells infiltrated perivascular area and variation in muscle fiber size and central nucleation were seen, indicating the presence of myositis. Medium-sized artery showed stenosis and occlusion of lumen with fibrinoid necrosis, indicating necrotizing vasculitis. No formation of granuloma was seen.

**Figure 2:**
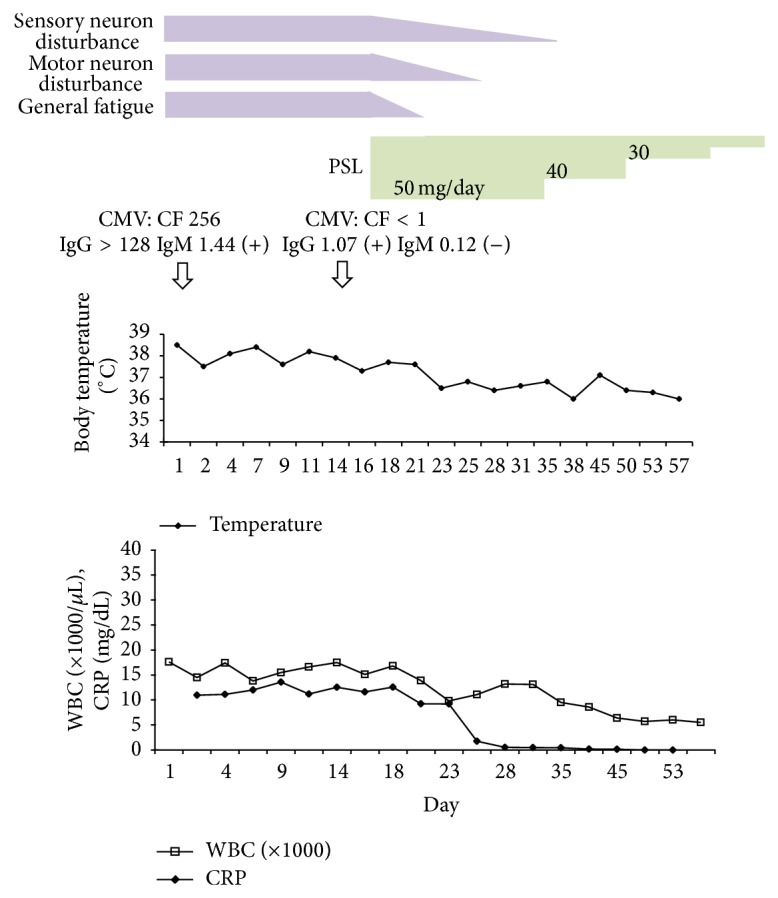
Clinical course of this case. After the diagnosis was made as PAN and CMV infection, he was given 50 mg of prednisolone and his symptoms were improved promptly and PSL dose could be reduced.

A 77-year-old man complained ofsore throat, cough, and low-grade fever. Due to the long duration of thefever, he was admitted to a nearby hospital to determine the reason for the fever. However, despitelaboratory and imaging examinations, the cause of the prolonged fever remained elusive.During 2 months of hospitalization, bilateral lower leg edema was noted, which gradually worsened until the patient could no longer walk unassisted. He was then transferred to our hospital for further examinations. He was slightly febrile (37.6°C) and had bilateral lower leg edema. He had also lost 7 kg over the last 3 months. Neurological examination showed muscle weakness of theright wrist extensor and flexor and left tibialis anterior hamstrings. There was hypoesthesia on both lower extremities and paresthesia in the right hand. Patellar and calcaneal tendon reflexes were absent. Laboratory investigations revealed an erythrocyte sedimentation rate of 65 mm/h, white blood cell count of 18,300/*μ*L, red blood cell count of 4.07 × 10^6^/*μ*L, hemoglobin of 11.5 g/dL, and platelets at 59.6 × 10^4^/*μ*L. Liver and renal function wasnormal. C-reactive protein was elevated to 15.67 mg/dL reflecting the presence of inflammation. Serological tests for anti-nuclear antibody, rheumatoid factor, MPO-ANA, PR3-ANCA, syphilis, viral hepatitis B, hepatitis C, parvovirus B19, HIV, and EB virus were all negative. However, serum CMV IgM by enzyme-linked immunosorbent assay (ELISA) was positive at admission. Although serum CMV IgG by complement fixation test (CF) was not examined before admission, the value of serum CMV IgG at admission showed more than four times elevation compared with that of 2 weeks after. Urinalysis, chest X-ray, and chest and abdominal CT were normal. In MRI, there were no sign**s** of aneurysm or arterialstenosis. However, electromyography revealed marked slowing of sensory and motor conduction velocities of the median and tibial nerves and demyelinating changes at distal extremities consistent with a diagnosis ofcombined mononeuritis multiplex and myopathy. A biopsy of the left biceps muscle was performed, revealing necrotizing vasculitis with fibrinoid necrosis (Figure 1). Considering all the data together, we diagnosed him as having PAN with active CMV infection according to diagnostic criteria, although there were few physical and laboratory findings that supported the presence of viral infection. The patient was given 50 mg of prednisolone (1 mg/kg/day) orally. His symptoms of fever, general fatigue, muscle weakness, and motor and sensory disturbance promptly improved and the PSL dose could be tapered (Figure 2). The patient left hospital in good condition when the PSL dose was down to 30 mg daily, which was further tapered off in the outpatient clinic.

## 3. Discussion

Vasculitides secondary to viral infections are well known, especially caused by HBV or HCV [3]. HBV infection is closely associated with the pathogenesis of PAN [4], so much so that it is one of the diagnostic criteria proposed by the American College of Rheumatology. Vasculitides associated with CMV infection have also been described but are mainly seen in immunocompromised patients, such as those infected with HIV. Their occurrence in healthy people seems to be rare [3]. Because vasculitides related to CMV infection are often life-threateningin immunocompromised patients, prompt intravenous antiviral treatment is recommended and the use of corticosteroid and/or immunosuppressants is discouraged for patients who are already immunosuppressed [3]. To the best of our knowledge, there have only been 3 reported cases of PAN suspected of being caused by CMV infection [5–7]. These 3 patients were treated with corticosteroids and/or antiviral drugs without additional immunosuppressants. This was different from consensus standard therapy for PAN. Similar to these previous cases, our patient was also treated with corticosteroid alone which led to rapid improvement of clinical symptoms and no relapse. Mayer et al. referred to the effectiveness of less aggressive treatment without immunosuppressants for ANCA-associated vasculitis induced by viral infection [7]. The clinical course of our case is consistent with their finding that patients with CMV-associated vasculitis do not require the aggressive therapy recommended as standard treatment for vasculitides. In our case, theinitial symptoms were fever, cough, and sore throat. At first, it was suspected that the patient was suffering from some sort of infection. This led to the serendipitous discovery of active CMV infection. In this regard, it should be noted that a patient who only shows symptoms that strongly suggest the presence of vasculitis at the first visit should be examined for the occurrence of underlying infection that could be involved in the pathogenesis of the vasculitis.

The limitation of our case is that we could not completely rule out the possibility of a flare of CMV induced by vasculitis because we had no definite serological data that indicated he was affected with CMV when he noticed initial symptoms such as sore throat, cough, and low-grade fever.

In conclusion, our case highlights the importance of CMV infection as one of the possible etiologies of vasculitis. Identifying the presence of an infection would also be important in terms of treatment selection. Less intensive therapy, such as corticosteroids in combination with antiviral agents, might be considered in patients with PAN associated with CMV infection.
